# Marker-assisted sex differentiation in date palm using simple sequence repeats

**DOI:** 10.1007/s13205-012-0052-x

**Published:** 2012-03-06

**Authors:** Khaled Elmeer, Imene Mattat

**Affiliations:** Genetic Engineering Department, Biotechnology Centre, Doha, Qatar

**Keywords:** *Phoenix dactylifera*, Sex identification, Sex marker, SSR, Microsatellite

## Abstract

Microsatellite markers containing simple sequence repeats (SSRs) are a valuable tool for genetic analysis. Our objective was to identify microsatellite markers that could be used to differentiate between male and female date palm (*Phoenix dactylifera*). The date palm is a dioecious plant whose sex cannot be determined until it reaches a reproductive age between 5 and 10 years. An early selection and/or differentiation of young seedlings into males and females could enhance breeding and assist research programs for genetic improvements of the date palm. Here, we report on the use of microsatellites for determining the sex of immature date palm. Using 14 microsatellite primer pairs with 129 date palm leaves and tissue culture samples from 34 cultivars which represent the major date palm diversity of Qatar, 254 microsatellite loci were detected, of these, 22 microsatellite loci could be used to identify 9 out of 12 male date palm samples (75%). The data also indicated that the heterozygous allele with the size 160/190 produced by the primer mPdCIR048 reoccurred 4 times exclusively in the 12 individual male samples but not in any of the 117 female date palm samples tested, and hence it is a promising candidate marker to detect male sex in date palm. Principal coordinate analysis (PCoA) of 12 male samples with 7 female Khasab cultivars produced 2 autonomous groups of males and females and similar results were observed with 13 female Shishi cultivars. Our results suggest that the SSR markers described here have potential in sex identification of date palm.

## Introduction

Palms (*Arecaceae*) are a particularly interesting family for the study of dicliny (separate male and female trees), as they display great diversity in their reproductive morphology, with more than 85% of the palm genera having single sex flowers (Dransfield et al. 2008). Historically, breeding programs to maintain genetic diversity have not been employed because there is no easy and accurate way to distinguish between male and female plants prior to first flowering, which occurs between 5 and 8 years after planting (Aberlenc-Bertossi et al. 2011; Bendiab et al. 1993). Date palm progenies consist of male and female individuals in equal proportions, which has led to the hypothesis that sex is determined genetically (Daher et al. 2010). Based on cytological studies with chromomycin staining, Siljak-Yakovlev et al. (1996) proposed the existence of sex chromosomes in the date palm. However, neither the gene associated with sex determination has been reported to date nor has the process of developmental arrest in sterile sex organs been studied in detail. Siljak-Yakovlev et al. (1996) also illustrate two other important points in understanding sex determination in dioecious species of plants. First, there are often no obvious cytological or genetic differences between male and female plants, and, second, it is often difficult to study the genetic or molecular basis of sex determination in many species of monoecious or dioecious, agronomically important plants simply because of their longevity.

A severe fusarium wilt of date palm, *Fusarium oxysporum*, recently destroyed date palms throughout Africa. The problem was exacerbated by the lack of natural genetic diversity in date palm populations, which may be overcome by introducing genetic variability into populations (especially for traits which confer disease resistance). The ability to type the sex of seedlings would speed this otherwise lengthy process (Juarez and Banks 1998).

In recent years, there have been serious efforts to understand the basis of sex determination in date palm and to develop methods of identifying the gender at an early stage using isozymes (Torres and Tisserat 1980), peroxydases (Majourhat et al. 2002), and molecular marker tools using random amplified polymorphic DNA (RAPD) (Moghaieb et al. 2010).

In this paper, we have attempted to identify sex-specific DNA markers for date palm cultivars using a microsatellite molecular technique. Such a technique would not only facilitate the identification and selection of good male pollinators for use in breeding programs but could also be used to select date palm with characteristics of high fruit yield, and an improved physical and chemical characteristic of the fruits.

## Materials and methods

### Plant material

Leaves and tissue culture samples from 117 female and 12 male date palms, comprising 34 cultivars, were collected from different locations in Qatar (Table 1). These cultivars represent the diversity of date palm genotypes in the Qatari date palm plantation. Young leaves from mature trees, randomly sampled, were collected and stored at −80 °C until DNA extraction.

**Table 1:** Cultivars and tissue culture samples of date palm (*Phoenix dactylifer)* from Qatar used in this study

Cultivar	Location				Tissue culture
	North	East	West	South	
Khalas	7	5	2	3	1
Shishi	2	5	2	3	1
Barhi	2	3	1	3	1
Khnaizi	1	4	2	3	1
Hillali	1	3	2	1	1
Khasab	1	3	1	2	0
Gar	2	2	0	0	1
Jabri	1	1	1	0	1
Lulu	0	2	2	0	0
Rzaiz	0	0	0	3	1
Nabetseaf	1	1	1	0	0
Shahil	1	0	1	1	0
Shabishi	0	1	2	0	0
Sukari	0	1	1	1	0
Iraqi	0	0	1	1	0
Deqlah	0	0	0	1	0
Gher	0	1	0	0	0
Marzban	1	0	0	0	0
Tanazel	0	0	1	0	0
Abumaan	0	0	0	0	1
Farthabaid	0	0	0	0	1
Zamli	0	0	0	0	1
Kathrawi	0	0	0	0	1
JeshRamli	0	0	0	0	1
Nawader	0	0	0	0	1
Hiri	0	0	0	0	1
Madayen	0	0	0	0	1
Namshi	0	0	0	0	1
Ghanami	0	0	0	0	1
UmDehan	0	0	0	0	1
Madjoul	0	0	0	0	1
Saaqi	0	0	0	0	1
FemaleR	0	0	1	0	0
FemaleY	0	0	1	0	0
Male	0	4	5	3	0

A total of 129 date palm samples representing 34 cultivars were collected

### DNA extraction

The frozen young leaf tissues were cleaned carefully with distilled water to remove the waxy layer and then 1 g of each leaf sample was cut into small pieces and grounded using liquid nitrogen into a fine powder. The DNA was extracted using the DNeasy Plant Maxi kit protocol (QIAGEN), following the manufacturer’s instructions outlined in the DNeasy Plant Handbook. The DNA samples obtained were quantified using NanoDrop^®^ ND 1000 Spectrophotometer (Thermo Fisher Scientific) and the quality was determined by electrophoresis of DNA samples (2 μL) loaded on 0.85% agarose gels and separated at 100 V for 30 min, following which the gel was stained with ethidium bromide and viewed under UV transilluminator.

### Microsatellite amplification

Fourteen labeled primer pairs as described by Billotte et al. (2004) were synthesized by Applied Biosystems (Belgium, Life Technologies Europe BV) and are presented in Table 2. A polymerase chain reaction (PCR) was performed using 25 μL of a reaction mixture containing 2 μL (5 ng) of total genomic DNA, 12.5 μL of AmpliTaq Gold^®^ 360 Mastermix (Applied Biosystems), 1 μL (5 pmol/μL) of forward primer (labeled), and 1 μL of reverse primer in addition to 8.5 μL of nuclease free water. Amplification was carried out in a Veriti 96 Well Fast Thermal cycler (Applied Biosystems) under the following conditions: initial denaturation at 95 °C for 10 min, 35 cycles (denaturation at 95 °C for 30 s, annealing temperature depending on primer for 30 s, and extension at 72 °C for 1 min), and final extension at 72 °C for 7 min.

**Table 2:** Forward and reverse microsatellite primers and their *T*_m_ used in this study (Billotte et al. 2004)

No.	Primer code	Repeat motif	Primer sequences (5′–3′)	Optimal *T*_m_ ( °C)
1	mPdCIR010	(GA)_22_	F: ACCCCGGACGTGAGGTG	55.9
			R: CGTCGATCTCCTCCTTTGTCTC	
2	mPdCIR015	(GA)_15_	F: AGCTGGCTCCTCCCTTCTTA	51.6
			R: GCTCGGTTGGACTTGTTCT	
3	mPdCIR016	(GA)_14_	F: AGCGGGAAATGAAAAGGTAT	51.7
			R: ATGAAAACGTGCCAAATGTC	
4	mPdCIR025	(GA)_22_	F: GCACGAGAAGGCTTATAGT	49.3
			R: CCCCTCATTAGGATTCTAC	
5	mPdCIR032	(GA)_19_	F: CAAATCTTTGCCGTGAG	51.5
			R: GGTGTGGAGTAATCATGTAGTAG	
6	mPdCIR035	(GA)_15_	F: ACAAACGGCGATGGGATTAC	53.9
			R: CCGCAGCTCACCTCTTCTAT	
7	mPdCIR044	(GA)_19_	F: ATGCGGACTACACTATTCTAC	51.7
			R: GGTGATTGACTTTCTTTGAG	
8	mPdCIR048	(GA)_32_	F: CGAGACCTACCTTCAACAAA	51.4
			R: CCACCAACCAAATCAAACAC	
9	mPdCIR057	(GA)_20_	F: AAGCAGCAGCCCTTCCGTAG	55.4
			R: GTTCTCACTCGCCCAAAAATAC	
10	mPdCIR070	(GA)_17_	F: CAAGACCCAAGGCTAAC	48.7
			R: GGAGGTGGCTTTGTAGTAT	
11	mPdCIR078	(GA)_13_	F: TGGATTTCCATTGTGAG	49.6
			R: CCCGAAGAGACGCTATT	
12	mPdCIR085	(GA)_29_	F: GAGAGAGGGTGGTGTTATT	50.4
			R: TTCATCCAGAACCACAGTA	
13	mPdCIR090	(GA)_26_	F: GCAGTCAGTCCCTCATA	48.6
			F: GCAGTCAGTCCCTCATA	
14	mPdCIR093	(GA)_16_	F: CCATTTATCATTCCCTCTCTTG	51.8
			R: CTTGGTAGCTGCGTTTCTTG	

### SSR fragment analysis

Simple sequence repeats (SSRs) were screened on a 3130 Genetic Analyzer (Applied Biosystems) by running 1 μL of PCR product mixed with 10 μL Hi-Di formamide and 0.3 μL GS500LIZ followed by denaturation at 95 °C for 3 min. The sample was then kept on ice for genotyping in a 3130 Genetic Analyzer. Automatic genotyping and allele scoring were performed by the GeneMapper^®^ software v4.0 (Applied Biosystems).

### Data analysis

The data were analyzed with PowerMarker software v3.0 (Liu and Muse 2005) to determine the percentage of heterozygosity, major allele frequency, number of alleles, gene diversity, and polymorphic information content (PIC).

The principal coordinate analysis (PCoA) of the male date palm with Shishi and Khasab cultivars was analyzed and drawn using PAST software v1.91 (Hammer et al. 2001) based on the Hamming distance measures by convex hulls.

## Results and discussion

The microsatellites examined were highly polymorphic, possessing a great number of alleles. A total of 124 alleles with a mean of 8.86 alleles per locus were scored, however, the number of alleles varied from 3 using primer mPdCIR090 to 13 using primers mPdCIR010 and mPdCIR078 (Table 3). The number of alleles per locus detected in this study was higher than those scored by Zehdi et al. (2004) who recognized 7.14 alleles per locus when examining 46 Tunisian date palm accessions using 14 microsatellite loci. On the other hand, Elshibli and Korpelainen (2007) identified 21.4 alleles per locus, which is more than the number of alleles per locus detected in this study. This may be a result of using a greater number of microsatellite loci (16) in addition to using different genotypes—68 Sudan and Morocco date palm accessions.

**Table 3:** Genetic diversity information by locus

Primer code	Allelic range	Major allele frequency	Genotype no.	Allele no.	Gene diversity	Heterozygosity	PIC
mPdCIR010	120–162	0.29	31.00	13.00	0.83	0.95	0.81
mPdCIR015	120–140	0.17	36.00	11.00	0.88	0.89	0.87
mPdCIR016	130–138	0.47	12.00	5.00	0.68	0.55	0.64
mPdCIR025	199–231	0.65	12.00	8.00	0.52	0.45	0.47
mPdCIR032	288–302	0.47	17.00	8.00	0.71	0.78	0.67
mPdCIR035	181–199	0.41	15.00	8.00	0.70	0.74	0.64
mPdCIR044	288–302	0.39	13.00	7.00	0.71	0.10	0.67
mPdCIR048	160–192	0.42	16.00	10.00	0.70	0.39	0.65
mPdCIR057	256–270	0.66	9.00	6.00	0.51	0.48	0.46
mPdCIR070	182–206	0.38	16.00	11.00	0.73	0.58	0.69
mPdCIR078	118–152	0.21	31.00	13.00	0.86	0.60	0.85
mPdCIR085	160–182	0.25	26.00	9.00	0.83	0.90	0.81
mPdCIR090	144–158	0.52	3.00	3.00	0.62	0.00	0.55
mPdCIR093	153–183	0.42	17.00	12.00	0.51	0.44	0.47
Mean		0.47	18.14	8.86	0.70	0.56	0.66

*PIC* polymorphic information content

The 14 primers used in this study successfully produced clear amplified SSR bands with sizes ranging from 118 bp with primer mPdCIR078 to 302 bp with primers mPdCIR044 and mPdCIR032 (Table 3), similar to the results of Ahmed and Al-Qaradawi (2009) where the band sizes ranged from 100 to 300 bp.

Interestingly, the 14 microsatellite primers used with the 117 female and 12 male date palm samples used in this study formed 254 microsatellite loci with a mean of 10.4 per primer (Table 3). The highest was 36 different microsatellite loci scored with primer mPdCIR015, but only 3 different microsatellite loci were scored with primer mPdCIR090.

In a number of agriculturally important plants, such as kiwi fruit, date palm, hops, papaya, and pistachio, the females produce the commercial harvest, while in some others, such as asparagus, males provide the better quality produce. Identification of the sex of such plants at their early stage of growth can be of great economic potential. Moreover, studies on marker technology regarding dioecy in general would provide a better understanding of the developmental (Ainsworth et al. 1998) as well as evolution pathways of dimorphism (Charlesworth and Charlesworth 1978; Charlesworth 1996).

Three primers (mPdCIR035, mPdCIR044, and mPdCIR090) could not distinguish between male and female samples (Table 4) whereas the remaining 11 microsatellite primers identified 22 loci, in only the male date palm samples. Using these loci, 9 of 12 (75%) male plants tested were recognized. Moreover, 82% of those loci were heterozygous alleles (Table 4), which is in agreement with the finding of Al-Dous et al. (2011) who scanned 3.5 million SNP genotypes in the male and female genomes of date palm to identify polymorphisms that segregate with gender. They observed that all male genomes shared mainly the same heterozygous genotypes, whereas female genomes shared mainly the same homozygous genotypes, while primer mPdCIR010 detected only one heterozygous allele even as the remaining three alleles were homozygous (Table 4). Sexually antagonistic polymorphisms are polymorphisms in which the allele is advantageous to one sex but is deleterious to the other sex. In an influential paper, Rice (1984) hypothesized that such polymorphisms should be relatively common on the X chromosome (or on the W in female-heterogametic species) but relatively rare on the autosomes. Yet, Fry (2010) showed that there are plausible assumptions under which the reverse is expected to be true, and pointed out studies that gave evidence for sexually antagonistic variation on the autosomes. Although more work is needed to resolve the issue, it is premature to conclude that the X chromosome is a “hot spot” for the accumulation of sexually antagonistic variation.

**Table 4:** Twenty two loci hetero and homozygous allele (base pairs) markers that identify male date palm trees

Locus code	Male 1	Male 2	Male 4	Male 5	Male 6	Male 8	Male 10	Male 11	Male 12
mPdCIR010	126/128	–	–	–	144/144	–	–	122/122	134/134
mPdCIR015	–	–	124/126	–	130/134	–	–	–	128/134
mPdCIR016	–	–	–	–	–	–	–	–	130/134
mPdCIR025	–	–	–	213/229	–	–	–	–	–
mPdCIR032	–	–	290/294	–	294/298	300/300	–	–	–
mPdCIR048	–	160/190	160/190	–	–	160/184	160/192	160/190	160/190
mPdCIR057	–	–	–	–	–	–	–	–	260/266
mPdCIR070	–	–	–	–	–	–	–	198/204	–
mPdCIR078	–	122/140	–	–	–	–	122/140	–	134/142
mPdCIR085	–	–	–	–	160/162	–	–	–	–
mPdCIR093	–	163/175	–	–	169/175	163/175	–	–	–

Primers with code mPdCIR035, mPdCIR044, and mPdCIR090 could not differentiate male and female date palm samples due to no amplification of distinguished markers in male plants therefore have been excluded from the table. For males 3, 7, and 9, no distinguished loci were detected and hence these samples have been excluded from the table

For male 12, 6 markers were detected while just one specific marker was detected for male 1. However, for males 3, 7, and 9, no markers were detected (Table 4).

Male associated DNA fragments have previously been identified by random amplification of polymorphic DNA (RAPD) in many dioecious plants. Sakamoto et al. (1995) cloned a 730 bp long DNA fragment named MADCl (male associated DNA sequence) in *Cannabis sativa*. However, MADC1 does not include a long ORF and is not likely to correspond to a transcribed gene (Sakamoto et al. 1995). Using representational difference analysis, several male sex-specific restriction fragments in *Silene latifolia* have been isolated and cloned. These male-specific restriction fragments were found to be homologous to other sequences shared between male and female plants (Domison et al. 1996).

The mean of the gene diversity was 0.70 (Table 3), ranging from 0.51 for loci mPdCIR057 and mPdCIR093 to high diversity 0.88 for locus mPdCIR015, indicating that the Qatari date palm collection is characterized by a high degree of genetic diversity. This level of gene diversity is similar to 0.70 reported for the Tunisian date palm germplasm (Zehdi et al. 2004) and less than 0.853 reported for the Sudanese date palm (Elshibli and Korpelainen 2007). This high level of diversity is expected because of the unique mechanism responsible for generating SSR allelic diversity by replication slippage. Replication slippage is thought to occur more frequently than single nucleotide mutations and insertion/deletion events, which generate the polymorphisms detected by RAPD analysis (Powell et al. 1996).

The heterozygous allele sized 160/190 exhibited by primer mPdCIR048 (Table 4) seems to be a distinguishing marker for sex in date palm because it appeared 4 times in the 12 individual male date palm trees tested. At the same time, no sign of this marker was detected in 117 individual female date palm trees. The two following alleles sized 122/140 (exhibited by the primer mPdCIR078) and 163/175 (exhibited by the primer mPdCIR093), respectively, were repeated twice in the 12 individual male date palm tree tested. Again, there was no sign of these alleles in the 117 individual female date palm trees.

The data obtained from the 14 primers combinations enabled the samples of the two date palm cultivars Shishi and Khasab to be classified into the two groups according to their sex expression compared with male trees using principal coordinate analysis (PCoA) (Gower 1966) using the PAST software v1.91 (Hammer et al. 2001). The Hamming distance was chosen in preference to other distance measures, as it does not class a common absence of an allele as a shared characteristic. It was, therefore, judged to be most appropriate for the present study, which included highly polymorphic microsatellite data spanning two ploidy levels.

**Fig. 1:**
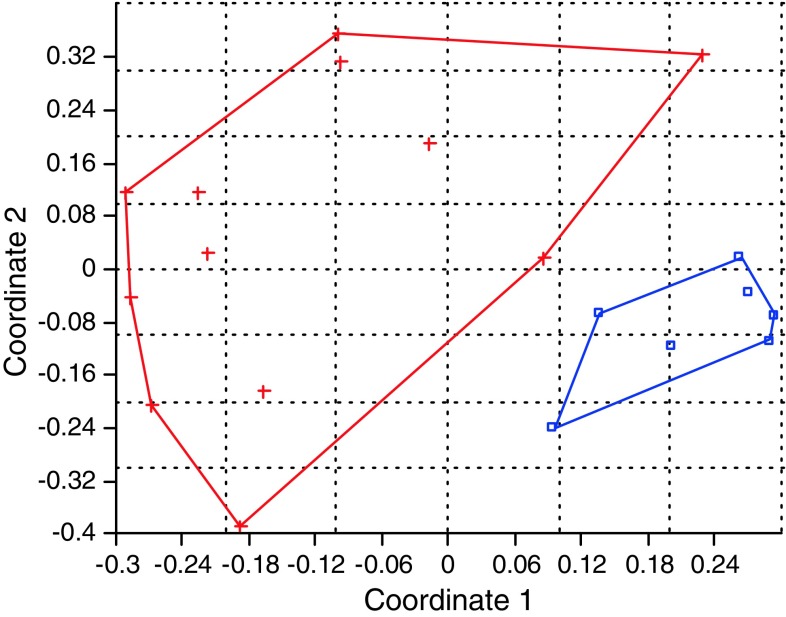
Scatter plot of the first and second principal coordinate analysis (PCoA) of 7 female Khasab cultivars (*blue*) and 12 male date palms (*red*) using Hamming distance measures on convex hulls based on the SSR obtained for the 14 primer combinations

**Fig. 2:**
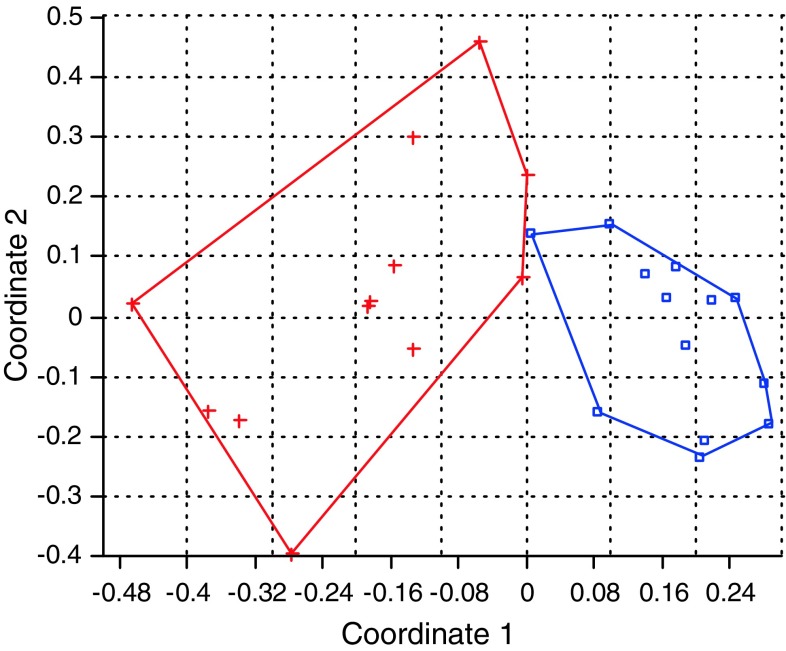
Scatter plot of the first and second principal coordinate analysis (PCoA) of 13 female Shishi cultivars (*blue*) and 12 male date palms (*red*) using Hamming distance measures on convex hulls based on the SSR obtained for 14 primer combinations

PCoA suggests two broad groups—one includes 7 female Khasab trees (Fig. 1) while the other is made up of 12 male date palm trees which are autonomous with 21% of the variation explained by the first axis and 17% by the second axis. The same two groups separate 13 female Shishi trees from the 12 male date palm trees (Fig. 2). There is a limited overlap between the two groups. Twenty percent of the variation is explained by the first axis while 13% of the variation is explained by the second axis.

Farmers are currently faced with distinguishing between cultivars propagated by seeds. The use of vegetative and flowering characteristics (Rhouma 1994) or the isozyme markers (Mohamed Ould et al. 2001) are less rewarding, since these traits take a long time—between 5 and 7 years—to become visible. Fortunately, the SSR alleles revealed were successfully used to molecularly discriminate between a nearly unlimited number of date palm cultivars. Compared to other reported date palm studies, the outcome in this study is more accurate than that observed using isoenzymes (Booij et al. 1995; Mohamed Ould et al. 2001) and plastid DNA haplotypes (Sakka 2003). Our data provide evidence that these powerful markers can be used as key identifiers of the sex in date palm materials.
